# The roles of exosomes in cancer drug resistance and its therapeutic application

**DOI:** 10.1002/ctm2.257

**Published:** 2020-12-21

**Authors:** Shiyu Li, Ming Yi, Bing Dong, Ying Jiao, Suxia Luo, Kongming Wu

**Affiliations:** ^1^ Department of Oncology Tongji Hospital of Tongji Medical College Huazhong University of Science and Technology Wuhan China; ^2^ Department of Molecular Pathology The Affiliated Cancer Hospital of Zhengzhou University & Henan Cancer Hospital Zhengzhou China; ^3^ Department of Medical Oncology The Affiliated Cancer Hospital of Zhengzhou University & Henan Cancer Hospital Zhengzhou China

**Keywords:** cancer, drug resistance, exosomes, therapeutic application

## Abstract

Exosomes are a category of extracellular vesicles with a size ranging from 40 to 160 nm, which can be secreted by multiple cells in the tumor microenvironment. Exosomes serve as communicators in regulating biological functions and pathological processes, including drug response. Through transporting the cargo such as protein or nucleic acid, exosomes can modulate drug sensitivity via multiple mechanisms. Additionally, exosomes can be deployed as a delivery system to treat cancer due to their high‐efficient loading capacity and tolerable toxicity. Recent studies have demonstrated the high efficacy of exosomes in cancer therapy. Herein, we conduct this review to summarize the mechanism of exosome‐mediated drug resistance and the therapeutic potential of exosomes in cancer.

## Graphical abstract

1. The mechanisms of exosome‐induced cancer drug resistance are elaborated.
2. The potential therapeutic application of exosomes is discussed in great detail.

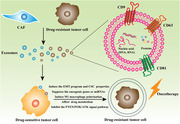

**Abbreviations**
ASM: acid sphingomyelinase
BB: blood/brain barrier
BC: breast cancer
CAAs: cancer‐associated adipocytes
CAFs: cancer‐associated fibroblasts
ceRNA: competing endogenous RNA
cGAS: cyclic GMP‐AMP synthase
CML: chronic myelogenous leukemia
CRC: colorectal cancer
CSCs: cancer stem cells
DCs: dendritic cells
DOX: doxorubicin
ELAVL1: ELAV like RNA binding protein 1
EMT: epithelial‐mesenchymal transition
ESCRT: endosomal sorting complex required for transport
EVs: extracellular vehicles
GBM: glioblastoma
GC: gastric cancer
GEM: gemcitabine
HCC: hepatocellular carcinoma
hnRNPA1: heterogeneous nuclear ribonucleoprotein A1
HSP 70: heat shock protein 70
MGMT: O‐6‐methylguanine‐DNA methyltransferase
miRISCs: miRNA loaded RNA‐induced silencing complexes
MM: multiple myeloma; mtDNA: mitochondrial DNA
NPC: nasopharyngeal cancer
NSCLC: non‐small cell lung cancer
NSKs: nanosponges and nanokillers
OXA: oxaliplatin
PDAC: pancreatic ductal adenocarcinoma
PDGFRβ: Platelet derived growth factor receptor β
PTX: Paclitaxel
SIRPA: Signal regulatory protein alpha
SMART‐Exos: synthetic multivalent antibodies retargeted exosomes
Sphk2: Sphingosine kinase 2
STING: stimulator of interferon genes
TAM: tumor‐associated macrophages
TDEs: tumor‐derived exosomes
TKIs: tyrosine kinase inhibitors
TME: tumor microenvironment
TRAIL: TNF‐related apoptosis‐inducing ligand
UCA1: urothelial carcinoma‐associated 1
ZEB1: zinc finger E‐box binding homeobox 1

## 1 INTRODUCTION

Extracellular vehicles (EVs) can be divided into four categories: exosomes, microvesicle, apoptotic bodies, and oncosomes.^1^ Thereinto, exosomes are formed through inward invagination of the endosomal membrane with a diameter ranging from 40 nm to 160 nm.^2^ But the latest standard proposed by minimal information for studies of extracellular vesicles 2018 (MISEV2018) has pointed out the term “small EVs” should be used when the particles are only characterized by size (<100 nm or <200 nm). The term “exosomes” is reserved to describe the EVs with particular biogenesis.^3^ Exosomes are identified by their hallmarks, such as CD9, CD63, CD81, ALIX, and heat shock protein 70 (HSP 70).^4^ Exosomes play a key role in intercellular communication involved in various biological functions and pathological processes.^5^ Moreover, cancer cells, immunocytes, adipocytes, fibroblasts, etc., all participate in tumor homeostasis, forming the so‐called tumor microenvironment (TME).^6^, ^7^ Exosomes secreted by these cells have an impact on oncogenesis, tumor progression, metastasis, as well as drug resistance.^8^

The intrinsic or acquired drug resistance results in the poor prognosis of cancer patients.^9^ The mechanisms attributing to drug resistance can be categorized as tumor cell factors, host factors, and host‐tumor interaction factors.^10^ Tumor cell factors consist of the elevated DNA damage repair, the presentation of epithelial‐mesenchymal transition (EMT) and cancer stem cells (CSCs), as well as the reduced intracellular drug concentration caused by drug transporters and intracellular drug‐metabolizing enzymes. The host factors refer to the genetic variants and interactions between different drugs. Tumor‐host interactions mean the interplay and the intercellular communication of various cells in the TME, which can be mediated by exosomes.^11^

Recently, there is an increasing number of studies exploiting the features of the high‐efficient loading capacity and transferring competence of exosomes to deliver drugs or functional RNAs.^12^, ^13^ Furthermore, exosomes can acquire tumor‐targeting ability by modifying their surface markers.^14^ Therefore, an exosomes‐based delivery method is regarded as a promising approach to treating cancer.

## 2 THE BIOGENESIS AND CARGO LOADING OF EXOSOMES

It is well accepted that exosomes are originated from the inward budding of the membrane of endosome, leading to the formation of multivesicular bodies, which eventually fuse with the plasma membrane to release exosomes.^15^ To begin with, cargos are recruited to multivesicular bodies. Furthermore, the endosomal sorting complex required for transport (ESCRT) is a critical protein complex participating in the protein loading via ubiquitin binding subunits.^16^ ESCRT‐associated proteins, such as ALIX and Tsg101, are also involved in the recruitment and loading of proteins even RNAs in the biogenesis of exosomes.^17^ In addition to the canonical ESCRT‐dependent pathway, there exists an ESCRT‐independent manner, in which ceramide plays a leading role. Ceramide may take part in sorting the bioactive molecules into exosomes and promoting domain‐induced budding. The ceramide‐rich domains curve spontaneously to form the invaginations, resulting in the generation of exosomes.^8^, ^18^ As for RNA, current research primarily focus on the sorting mechanism of miRNA. Apart from ESCRT proteins and ceramide, there are several other mechanisms concerning miRNA sorting. It is well known that RNA‐binding proteins can recognize the specific motif in the 3′ portion of miRNA to facilitate its loading into exosomes.^19^ The selection of miRNAs is also related to their 3′ end post‐transcriptional modifications. The miRNAs with uridylation are likely released to the extracellular environment via exosomes while those with 3′ end adenylation are inclined to retain into the cell.^20^ Furthermore, miRNA loaded RNA‐induced silencing complexes (miRISCs) have an influence on the process of miRNA sorting to exosomes. miRISCs exert their function by regulating the silencing process of RNA and affecting the intracellular locations of miRNA and miRNA‐repressible transcript.^21^ Although the knowledge on how lncRNA is loaded into exosomes is insufficient, they are identified to share a similar sorting mechanism with mRNA.^22^ In this process, RNA‐binding protein YBX1 can recognize the specific sequences and secondary structures in the 3′‐untranslated regions of mRNA or lncRNA.^23^ After the release, exosomes act through delivering the selected cargos. In the section below, we will discuss the role of exosomes in drug resistance according to the different categories of cargos.

## 3 THE ROLE OF EXOSOMES IN DRUG RESISTANCE

**FIGURE 1:**
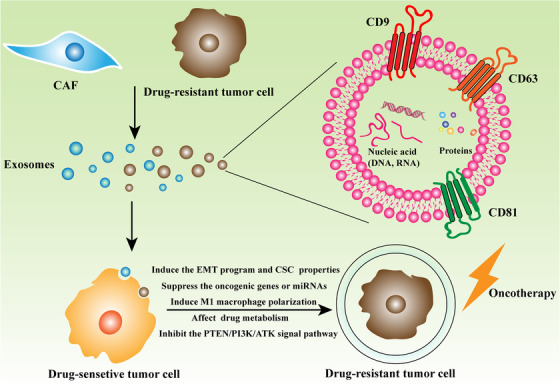
The mechanism by which exosomes confer sensitive tumor cell with the drug resistance phenotype. Exosomes mainly derived from CAF (cancer‐associated fibroblast) or drug‐resistant tumor cell can be effectively taken up by drug‐sensitive cell. Then, exosomal cargos, including nucleic acid (DNA and RNA) and proteins, indeed play an important part in transferring drug resistance phenotype to drug‐sensitive cell. Finally, drug‐sensitive cell acquires the capability to resist oncotherapy like chemotherapy or molecular target therapy

Exosomes can regulate drug resistance by directly interacting with drugs, changing the transcriptome of cancer cells as well as influencing the immune response. The distinct roles of exosomes in drug resistance depend on their contents, including nucleic acids, proteins, lipids, metabolites, and so on^2^ (Figure 1).

### 3.1 Exosomal nucleic acid cargo mediating drug resistance

#### 3.1.1 Exosomal miRNA

Recently, there is a growing number of studies about the roles that miRNAs play in drug resistance (Table 1). MiRNAs function through modulating protein level by specifically interacting with target mRNAs.^24^ In most cases, exosomal miRNAs induce therapeutic resistance. Exosomal miRNAs derived from drug‐resistant tumor cells can confer the resistant phenotype to sensitive ones. For example, exosomal miR‐100‐5p and miR‐222‐3p from non‐small cell lung cancer (NSCLC) cells promote drug resistance in recipient cells by targeting the mammalian target of rapamycin and suppressor of cytokine signaling 3, respectively.^25^, ^26^ CDK4/6 inhibitors are the breakthrough for the treatment of estrogen receptor‐positive breast cancer (BC) at present.^27^ Recently, a study revealed the mechanism of the acquired resistance to palbociclib (one of the CDK4/6 inhibitors). Here, miR‐432‐5p plays a key role in downregulating TGF‐β to increase CDK6 expression. It is exosomal miR‐432‐5p that transfers the resistant capability to adjacent cell populations. Intriguingly, this resistance can be reversed after a drug holiday, indicating that the reintroduction of CDK4/6 inhibitor is still effective after a certain withdrawal time.^28^ It is urgent to find whether such a reversible resistance exists in the majority of BC patients with the treatment of CDK4/6 inhibitor, which may favor patient management. In conventional chemotherapy of BC, tumor‐derived exosomal miRNAs also contribute to drug resistance. For instance, BC cells acquire resistance to docetaxel, epirubicin as well as gemcitabine (GEM) after the transfer of exosomes carrying miR‐1246, which targets cyclin‐G2.^29^

**TABLE 1:** The role of exosomal nucleic acid cargo in inducing therapy resistance

Cargo type	Cargo	Cancer type	Resistant therapy	Expression in drug‐resistant cancer	Exosome origin	Target of cargo	Ref.
miRNA	miR‐100‐5p	NSCLC	Cisplatin	Upregulation	Tumor	mTOR	25
	miR‐222‐3p	NSCLC	GEM	Upregulation	Tumor	SOCS3	26
	miR‐425‐3p	NSCLC	Cisplatin	Upregulation	Tumor	ATK1	46
	miR‐522‐3p	NSCLC	Gefitinib	Upregulation	Tumor	ATK	136
	miR‐522	GC	PTX, cisplatin	Upregulation	CAFs	ALOX15	30
	miR‐155	BC	PTX, DOX	Upregulation	Tumor	FOXO3	43
	miR‐432‐5p	BC	CDK4/6 inhibitor	Upregulation	Tumor	TGF‐β	28
	miR‐1246	BC	DTX, GEM, epirubicin	Upregulation	Tumor	CCNG2	29
	miR‐203a‐3p, miR‐195‐5p, miR‐9‐5p	BC	DOX, DTX	Upregulation	Tumor	ONECUT2	42
	miR‐196a	HNC	Cisplatin	Upregulation	CAFs	ING5, p27	31
	miR‐365	PDAC	GEM	Upregulation	TAMs	NA	37
	miR‐32‐5p	HCC	5‐FU, OXA, GEM, sorafenib	Upregulation	Tumor	PTEN	38
	miR‐92‐3p	CRC	5‐FU, OXA	Upregulation	CAFs	FBXW7, MOAP1	39
	miR‐21	OC	PTX	Upregulation	CAAs and CAFs	APAF1	32
	miR‐223	OC	Cisplatin	Upregulation	TAMs	PTEN	45
	miR‐770	BC	DOX	Downregulation	Tumor	STMN1	36
	miR‐128‐3p	CRC	OXA	Downregulation	Intestinal epithelial cells	Bmi1, MRP5	41
	miR‐7	GBM	TRAIL	Downregulation	MSCs	XIAP	48
	miR‐567	BC	Trastuzumab	Downregulation	Breast epithelial cells	ATG5	49
lncRNA	UCA1	NSCLC	Gefitinib	Upregulation	Tumor	FOSL2	50
	UCA1	OC	Cisplatin	Upregulation	Tumor	FOSL2	51
	PART1	EC	Gefitinib	Upregulation	Tumor	miR‐129	52
	lncARSR	RCC	Sunitinib	Upregulation	Tumor	miR34/449	54
	SBF2‐AS1	GBM	TMZ	Upregulation	Tumor	miR‐151a	56
	AFAP1‐AS1	BC	Trastuzumab	Upregulation	Tumor	AUF1	57
	H19	CRC	OXA	Upregulation	CAFs	miR‐141	53
	CCAL	CRC	OXA	Upregulation	CAFs	ELAVL1	58
CircRNA	CircNFIX	Glioma	TMZ	Upregulation	Tumor	miR‐132	60
	hsa_circ_0005963	CRC	OXA	Upregulation	Tumor	miR‐122	62
	Cdr1as	OC	Cisplatin	Downregulation	Tumor	miR‐1270	63
mRNA	MGMT	Glioma	TMZ	Upregulation	Reactive astrocyte	NA	64
	ZEB1	NSCLC	Cisplatin, GEM	Upregulation	Tumor	NA	67
DNA	Mitochondrial DNA	BC	Hormonal therapy	Upregulation	CAFs	NA	68

**Abbreviations**: ALOX15, arachidonate 15 lipoxygenase; APAF1, apoptotic peptidase activating factor 1; ATG5, autophagy‐related 5; AUF1, AU‐binding factor 1; BC, breast cancer; Bmi1, B lymphoma Mo‐MLV insertion region 1 homolog; CAAs, cancer‐associated adipocytes; CAFs, cancer‐associated fibroblasts; CCNG2, cyclin G2; CRC, colorectal cancer; DOX, doxorubicin; DTX, docetaxel; EC, esophageal cancer; EVAVL1, ELAV like RNA binding protein 1; FBXW7, F‐box and WD repeat domain containing 7; FOSL2, FOS like 2; FOXO3, forkhead box O3; GBM, glioblastoma; GC, gastric cancer; GEM, gemcitabine; HCC, hepatic carcinoma; HNC, head and neck cancer; ING5, inhibitor of growth protein 5; MOAP1, modulator of apoptosis 1; MRP5, multidrug resistance‐associated proteins 5; MSCs, mesenchymal stem cells; mTOR, mammalian target of rapamycin; NA, not acquired; NSCLC, non‐small cell lung cancer; OC, ovarian cancer; ONECUT2, one cut homeobox 2; OXA, oxaliplatin; PDAC, pancreatic ductal adenocarcinoma; PPARδ, peroxisome proliferator activated receptor δ; PTX, paclitaxel; RCC, renal cell carcinoma; SOCS3, suppressor of cytokine signaling 3; STMN1, stathmin 1; TAMs, tumor‐associated macrophages; TMZ, temozolomide; TRAIL, TNF‐related apoptosis‐inducing ligand; XIAP, X‐linked inhibitor of apoptosis.

Exosomal miRNAs derived from stromal cells (mainly are cancer‐associated fibroblasts [CAFs]) can also regulate the drug resistance of cancer cells. CAF‐derived exosomal miR‐522 is able to inhibit ferroptosis by repressing arachidonate lipoxygenase 15 and contribute to the depressing accumulation of cytotoxic lipid peroxides in gastric cancer (GC). Notably, paclitaxel (PTX) and cisplatin can promote the secretion of exosomal miR‐522, leading to the acquired resistance to chemotherapy. As for the mechanism, chemotherapy induces the upregulation of ubiquitin‐specific protease 7, which stabilizes heterogeneous nuclear ribonucleoprotein A1 (hnRNPA1), ultimately regulating the exosomal package of miR‐522.^30^ Besides, hnRNPA1 is identified to mediate miR‐196a packing into CAF‐derived exosomes in head and neck cancer, which contributes to intrinsic resistance to cisplatin by targeting inhibitor of growth protein 5 and p27.^31^ In ovarian cancer (OC) cells, exosomal miR‐21 derived from cancer‐associated adipocytes (CAAs) and CAFs is responsible for PTX resistance.^32^ Moreover, tumor‐derived exosomes (TDEs) can “educate” CAAs, in turn, to create a favorable microenvironment for tumor progression.^33^

In addition to directly regulating the expression of the drug‐resistant gene, exosomal miRNAs are able to affect the phenotype of chemotherapy resistance by changing the phenotype of tumor‐associated macrophages (TAMs). Macrophages are generally classified into two phenotypes: M1 populations (pro‐inflammation) and M2 populations (anti‐inflammation).^34^ Abundant M2 phenotype macrophages in the TME play a carcinogenic function of repressing anti‐cancer immune response while M1 ones exert the opposite function.^35^ In triple‐negative BC, ectopic expression of miR‐770 mediated by exosomes enhances doxorubicin (DOX) sensitivity via modulating apoptosis and TAM phenotypes. The restoration of stathmin 1, a target of miR‐770, can reverse the DOX resistance partially.^36^ In addition, TAMs can affect drug resistance through secreting exosomal miRNAs as well. For example, exosomal miR‐365 secreted by TAMs with M2 phenotype plays a cancer‐promoting part in contributing to the GEM resistance in pancreatic ductal adenocarcinoma (PDAC). Exosomal miR‐365 principally exerts its GEM‐resistant function in two ways, one of which is through impeding GEM activation by the upregulation of triphospho‐nucleotide pool and another is through inducing cytidine deaminase to make GEM inactivation.^37^

Exosomes with miRNAs can confer recipient cells with drug resistance competence through affecting EMT or CSC properties. In multidrug‐resistant HCC, exosomal transfer of miR‐32‐5p enhances the resistant capability of sensitive cells by regulating EMT and angiogenesis.^38^ In colorectal cancer (CRC), exosomal miR‐92‐3p and miR‐146a‐5p are related to chemoresistance coupled with the appearance of EMT or CSC properties^39^, ^40^ Nevertheless, miR‐128‐3p is identified as a tumor suppressor by repressing EMT and promoting intracellular chemotherapeutics accumulation in CRC. This is because miR‐128‐3p can impede oxaliplatin (OXA)‐induced EMT and hinder OXA efflux simultaneously through exosomes.^41^ In BC, miR‐203a‐3p, miR‐195‐5p, miR‐9‐5p, and miR‐155 are all reported to contribute EMT‐ and CSC‐associated chemoresistance.^42^, ^43^

Importantly, the PTEN/PI3K/ATK signal pathway is a pivotal pathway to modulate exosomal miRNAs‐induced drug resistance. PTEN is correlated with the reduction of cancer cells proliferation, invasion, and drug resistance by negatively regulating the PI3K/ATK pathway.^44^ In OC, exosomes enriched with miR‐223 are secreted from M2 macrophages together with enhanced drug resistance under the hypoxic circumstance. The PTEN/PI3K/AKT pathway is involved in the mechanism of resistance. Clinically, upregulated miR‐223 is also significantly related to the increased HIF‐1α expression and higher recurrence risk of OC.^45^ In NSCLC, exosomal miR‐425‐3p is found to mediate cisplatin resistance by targeting AKT1. Notably, cisplatin treatment will induce the miR‐425‐3p expression in the transcriptional level through Wnt/β‐catenin/c‐Myc pathway.^46^ However, it is reported that β‐elemene can reverse drug resistance via regulating the level of several miRNAs in exosomes such as miR‐34 and miR‐452. The upregulation of PTEN induced by β‐elemene plays a leading role in this process.^47^

However, there are still some miRNAs reversing drug resistance. MiR‐7 is identified as a natural sensitizer of TNF‐related apoptosis‐inducing ligand (TRAIL) and promotes apoptosis in TRAIL‐resistant glioblastoma (GBM) cells in an exosome‐dependent manner.^48^ Moreover, a low expression level of miR‐567 in HER‐2 positive BC is significantly related to the poor response to trastuzumab. Importantly, the miR‐567‐contained exosomes can be taken up by recipient cells, transforming the trastuzumab‐resistant phenotype into the trastuzumab‐sensitive one. Exosomal miR‐567 promoted trastuzumab sensitivity via downregulating the expression of autophagy‐related 5 that was responsible for the resistance through decreasing autophagy rate.^49^ Currently, HER‐2 is the only marker to identify patients who will benefit from HER‐2‐based treatment. Thus, it is important to find other novel potential biomarkers like miR‐567 for guiding the therapeutic decision and predicting the therapeutic response of HER‐2 positive BC patients.

It is well‐accepted that cancer cells can resist the drug‐induced toxicity. Numerous studies have reported the role of exosomes in promoting drug resistance. The exosomes derived from cancer cells or CAFs can “educate” recipient cells by intracellularly releasing the bioactive cargos then activating the specific pathways. Although the resistance‐related pathways mediated by exosomal miRNAs have been broadly investigated, the functions of exosomal miRNAs remain unclear yet owing to their tissue specificity and target gene diversity. In addition, inhibition of drug resistance‐induced miRNA cannot entirely abolish the resistance, indicating drug resistance is a complex process where other factors may be involved.^45^ Therefore, more efforts should be made for elucidating the molecular basis of drug resistance. One implication of the studies about the roles of miRNAs in drug resistance is to provide a therapeutic strategy to antagonize the miRNAs and inhibit the downstream signal pathways. Furthermore, exosomal miRNAs can be applied to select the drug‐sensitive patients and monitor the response to the treatment, which may have a better performance than circulating miRNAs due to the stability. Thus, another research priority is to identify the reliable exosomal biomarkers for diagnosis and prognosis prediction.

#### 3.1.2 Exosomal lncRNA

LncRNA transferred by exosomes usually serves as a competing endogenous RNA (ceRNA) of miRNAs to exert its function of modulating drug resistance. In NSCLC cells, lncRNA urothelial carcinoma‐associated 1 (UCA1) modulates gefitinib resistance through delivering exosomes. The recipient cells show gefitinib resistant‐phenotype, in which the expression of miR‐143 is decreased with the increased expression of its potential target, FOS like 2.^50^ In addition, lncRNA UCA1 induces cisplatin resistance in OC via the same pathway.^51^ Kang et al has reported that upregulated lncRNA PART1 was incorporated into exosomes to promote gefitinib resistance in esophageal squamous cell carcinoma. There is a clinical association between high exosomal PART1 level in serum and the poor response to gefitinib therapy. Mechanically, lncRNA PART1 is identified as a ceRNA of miR‐129 to promote Bcl‐2 expression.^52^ Moreover, lncRNA H19 and lncARSR are reported to reduce drug sensitivity by serving as a ceRNA of miR‐141 and miR‐34/449, respectively.^53^, ^54^ Temozolomide (TMZ) has been used as a first‐line drug for the treatment of GBM for over a decade. However, the acquired resistance limits the therapeutic effect of TMZ.^55^ Recently, TMZ resistance was found to be clinically associated with serum exosomal lncRNA SBF2‐AS1. Mechanically, SBF2‐AS1 enhances DNA double‐strand break repair, which is currently regarded as a leading cause of TMZ resistance, via serving as a ceRNA of miR‐151a.^56^ Although exogenous expression lncRNAs and anti‐miRNAs can both inhibit the downstream signal pathways to exert their anti‐drug resistance function, lncRNAs are more effective than the conventional anti‐miRNA approach.^51^ This phenomenon emphasizes the powerful therapeutic potential of exosomal lncRNA.

Exosomal lncRNA is also able to impact drug resistance by regulating RNA‐binding proteins. Transfer of exosomal lncRNA AFAP1‐AS1 is reported to be responsible for the shorter survival time of HER‐2 positive BC patients partially due to the trastuzumab resistance. Intriguingly, AFAP1‐AS1 induces the resistance to trastuzumab by upregulating HER‐2 expression in the translational level rather than the transcriptional level via binding to RNA‐binding protein, AU‐binding factor 1.^57^ LncRNA CCAL transported from CAFs to CRC cancer cells via exosomes leads to the acquisition of OXA resistance both in vitro and in vivo. Mechanically, CCAL facilitates β‐catenin expression by post‐transcriptional regulation in an ELAV like RNA binding protein 1 (ELAVL1)‐dependent manner, which stabilizes β‐catenin mRNA through binding the AU‐rich elements.^58^ Since the exosomal lncRNA can affect the drug resistance via an RNA‐binding protein‐dependent manner, a novel strategy of suppressing the expression of RNA‐binding protein will be attractive. Nonetheless, whether this strategy generates the additive effect remains to be further explored when the lncRNAs are blocked. Although exosomes can protect lncRNAs from degradation in the circulation, exosomal lncRNAs only accounts for 20.19% of exosomal RNA.^59^ Besides, the current technique of exosome isolation and enrichment is low yield, therefore further optimization is required. It is doubtful whether the relatively low concentration of exosomal lncRNAs in biofluids is feasible to serve as predictors for drug resistance.

#### 3.1.3 Exosomal circRNA

In the past few years, with the high‐throughput sequencing technology growing popular, researchers can detect the expression of circRNA. It is well known that circRNA can induce drug resistance by serving as miRNA sponges. Through sponging miR‐132 that represses glioma progression by inhibiting the invasion as well as migration and facilitating cell apoptosis of tumor cells, exosomal CircNFIX confers the ability of TMZ resistance to recipient cells in glioma.^60^ Some ATP‐dependent drug efflux pumps like MRP play a pivotal role in promoting drug resistance. With the increase of intracellular ATP, the pumps can acquire more energy to transport drugs out of cells, which can protect the cell from cytotoxicity. However, the approaches targeting the drug efflux pumps like P‐glycoprotein inhibitor and RNA interference haven't achieved the desired effects.^61^ Thus, Wang et al focused on reducing the energy source of drug efflux pumps rather than inhibiting them directly. The authors found the circular RNA hsa_circ_0005963 packed into exosomes from OXA‐resistant CRC cells is delivered to sensitive ones, thereby boosting glycolysis to attenuate the drug sensitivity. Mechanism exploration indicated that hsa_circ_0005963 identified as a sponge of miR‐122 upregulated the expression of pyruvate kinase M2, through which the sensitive cells will be progressively transformed into chemo‐resistant cells.^62^ Nevertheless, the therapeutic effect of reducing the ATP of drug efflux pumps should be further evaluated compared with the direct inhibition of drug efflux pumps.

On the contrary, exosomal circRNA can serve as a sensitizer of chemotherapy. For instance, the expression of exosomal circular RNA Cdr1as in serum is inversely relevant to the cisplatin‐resistant phenotype in OC patients. The experimental study proves that ectopic expression of Cdr1as reduces cellular proliferation and enhances the cisplatin‐induced cell apoptosis in OC cells. The underlying mechanism is that Cdr1as can repress cisplatin resistance by downregulating miR‐1270, thereby increasing the suppressor of cancer cell invasion.^63^

#### 3.1.4 Exosomal mRNA and DNA

Exosomal mRNA and DNA are fields where few researchers are involved, which also play key roles in inducing drug resistance. In neurologic tumors, exosomal O‐6‐methylguanine‐DNA methyltransferase (MGMT) mRNA plays a key role in TMZ resistance. For example, normal human astrocyte can protect glioma cells from TMZ‐induced apoptosis via transferring exosomal MGMT mRNA both in vitro and in vivo.^64^ In another study about GBM, high levels of MGMT mRNA and Nestin with low expression of Transglutaminase 2 were discovered to be relevant to the appearance of CSC features, contributing to TMZ resistance.^65^ Zinc finger E‐box binding homeobox 1 (*ZEB1*) is regarded as a key transcription factor of EMT, which is also involved in drug resistance.^66^ Exosomal ZEB1 mRNA induces the mesenchymal phenotype of recipient cells, thereby leading to the chemoresistance in NSCLC.^67^ As for exosomal DNA, there is a study about the relation between mitochondrial DNA (mtDNA) and hormonal therapy resistance in BC. CAF‐derived exosomes from hormonal therapy‐resistant BC patients or xenograft models contain the whole mtDNA that contributes to this phenotype. Co‐cultured with exosomal mtDNA, BC cells acquire resistant phenotype with the resurgence of dormant CSCs caused by the increased oxidative phosphorylation ability.^68^ However, the way by which the special mitochondrial genome is loaded, released, endocytosed, and incorporated within the mitochondria is unclear.

### 3.2 Exosomal protein cargo mediating drug resistance

Protein incorporated into exosomes has a marked influence on chemotherapy resistance. According to the different mechanisms of action on inducing drug resistance, exosomal proteins can be mainly divided into enzymes, transcription factors, membrane proteins or receptors, and secretory proteins like cytokines.

The exosomal enzyme is one of the categories of the exosomal drug resistance‐associated protein. Generally, enzymes play a crucial role in physiological or pathological processes by catalyzing the formation of downstream products. Heparanase, an endo‐β‐D‐glucuronidase, can cleave heparan sulfate chains. Recently, Bandari et al discovered that the expression of heparanase was elevated in the MM cells that survive chemotherapy. The mechanism is that exosomes loaded with heparanase promote the degradation of heparan sulfate, resulting in extracellular signal‐regulated kinase (termed ERK) signaling activating and syndecan‐1 shedding.^69^ Exosomes are enriched in ceramide, which is formed after the hydrolytic removal of the phosphocholine of sphingomyelin by sphingomyelinases.^70^ Exosomal acid sphingomyelinase (ASM) is found to confer the resistance to melphalan or bortezomib to sensitive multiple myeloma (MM) cells.^71^ It is likely that exosomal ASM mediates the intercellular communication between drug‐resistant MM cells and drug‐sensitive ones by increasing the release of drug resistance‐associated exosomes. Importantly, amitriptyline, which is usually applied to alleviate the pain of MM patients, is found to inhibit ASM expression resulting in the re‐sensitivity to melphalan or bortezomib.^71^ Given that the studies about lipid metabolism pathways in exosome‐induced drug resistance are rare, it is interesting to perform lipidomic analysis to identify the differential expression of exosomal lipids in the future. Notably, the diagnostic or prognostic values of identified exosomal lipids should be further verified in a large number of clinical studies.

Transcription factors are also a crucial portion of exosomal drug resistance‐associated protein. Javidi‐Sharifi et al reported that fibroblast growth factor 2 secreted from bone marrow stromal cells was packed into exosomes that were endocytosed by leukemia cells, which subsequently acquired the resistant ability to tyrosine kinase inhibitors (TKIs).^72^ Now that the resistance to TKIs is frequently observed in the clinic, fibroblast growth factor 2 inhibitor may be a promising strategy to improve the durable response to TKIs. In addition, the contents of exosomes from 5‐FU‐resistant CRC cells are identified as p‐STAT3 and Glutathione S‐Transferase Pi 1, endowing sensitive cells with the capability of resisting to 5‐FU. In this process, p‐STAT3 acts as a transcription activator and makes a real difference through the caspase cascade.^73^

Moreover, exosomal membrane protein or receptors play a pivotal role in inducing drug resistance by activating or inhibiting specific signal pathway. Exosomal caveolin‐1 can elicit NF‐κB cascade to affect EMT and CSC phenotype, consequently contributing to chemoresistance in castration‐resistant prostate cancer.^74^ B‐Raf proto‐oncogene, Serine/Threonine kinase (termed BRAF) belongs to the RAF family of serine/threonine protein kinases, whose mutation accounts for 40‐60% of cutaneous melanomas.^75^ Although BRAF inhibitor including dabrafenib and vemurafenib has been applied to clinical therapy, the relapse rate is still high. Thus, finding a reliable biomarker to identify patients with preexisting intrinsic resistance to BRAF inhibitor can significantly minimize clinical distress and loss, which remains a critical challenge. A study ascribes the recrudesce to a drug‐resistant driver platelet derived growth factor receptor β (PDGFRβ). The BRAF inhibitor‐resistant melanomas cells alter the phenotype of neighboring sensitive ones by delivering exosomal PDGFRβ.^76^ Exosomal PDGFRβ will be a promising biomarker for monitoring the therapeutic response thereby facilitating timey clinical decision.

The research also focus on the role of secretory proteins in the process of drug resistance. However, secretory proteins within exosomes possess distinct function in affecting drug resistance. For instance, exosomal Wnts from CAFs can contribute to chemotherapy resistance by dedifferentiation, through which CRC cells acquire CSC‐like phenotype to resist chemotherapy.^77^ Moreover, exosomal cytokines like Activin‐A, IL‐6 as well as G‐CSF are proved to confer chemoresistance to recipient cells, which is involved in a similar mechanism like exosomal Wnts.^78^ Subsequent research priority is to find specific molecules, which regulate the secretion of these exosomal cytokines.

## 4 INHIBITING THE FORMATION AND RELEASE OF EXOSOMES

Since exosomes participate in the process of drug resistance through various mechanisms, a strategy based on inhibiting the formation and release of exosomes for overcoming drug resistance has emerged. After the first report that neutral sphingomyelinase inhibitor GW4869 could inhibit the release of exosomes via decreasing the formation of ceramide, GW4869 has been gradually applied to exosomes research.^79^ It is found that GW4869 can reverse the exosome‐induced malignant biological properties of NPC cells, like migration, proliferation, and DOX resistance accompanied by the EMT process.^80^ Likewise, while using the anti‐PD‐L1 antibody, inhibiting the secretion of exosomal PD‐L1 by GW4869 shows an additive therapeutic efficacy in overcoming the resistance.^81^ Recently, the ability of novel agents to counter the formation and release of exosomes through other mechanisms has been discovered, which can be employed for reducing drug resistance. It is well known that lipid rafts play an important part in modulating the production and release of exosomes. A study shows that γ‐Tocotrienol can directly disrupt the integrity of lipid rafts further indirectly repressing the exosomal heregulin‐mediated antitumor drugs resistance, which can activate HER3 and HER4 in BC cells.^82^ As the critical biomarker of exosomes, the expression of CD9 and CD63 can be inhibited by plant defensin PvD1 in BC cells, subsequently affecting the formation of exosomes. PvD1 is able to bind to the mature exosomes in the extracellular environment as well, highlighting its potential therapeutic capability in antagonizing exosomes‐mediated malignant behaviors of cancer.^83^ However, the study didn't elucidate the mechanism of the aberrant expression of CD9 and CD63 caused by PvD1. In addition, bisindolylmaleimide‐I or chloramidine can also significantly improve the efficacy of anti‐tumor therapy by impeding the release of exosomes.^84^ Further exploration reveals that bisindolylmaleimide‐I, a protein kinase C inhibitor, prevents the externalization of phosphatidylserine, which is a known mechanism of driving exosomes release. At the same time, chloramidine can affect peptidylarginine deiminase activation to reduce the cellular exosomes release.^85^, ^86^ The gradually expanded repertoire of exosome inhibiting agents has a promising future to overcome the drug resistance. More emphasis should be placed on whether the emerging agents have an influence on cell viability or generate other side effects. According to the biosynthesis of different cargos within exosomes, the agents should be individually and synergistically tested to find a proper approach to optimize the therapeutic effects. It is preferable to use the EVs inhibitors instead of exosomes inhibitors due to the complicated intercellular communication.

## 5 POTENTIAL THERAPEUTIC APPLICATION OF EXOSOMES

**FIGURE 2:**
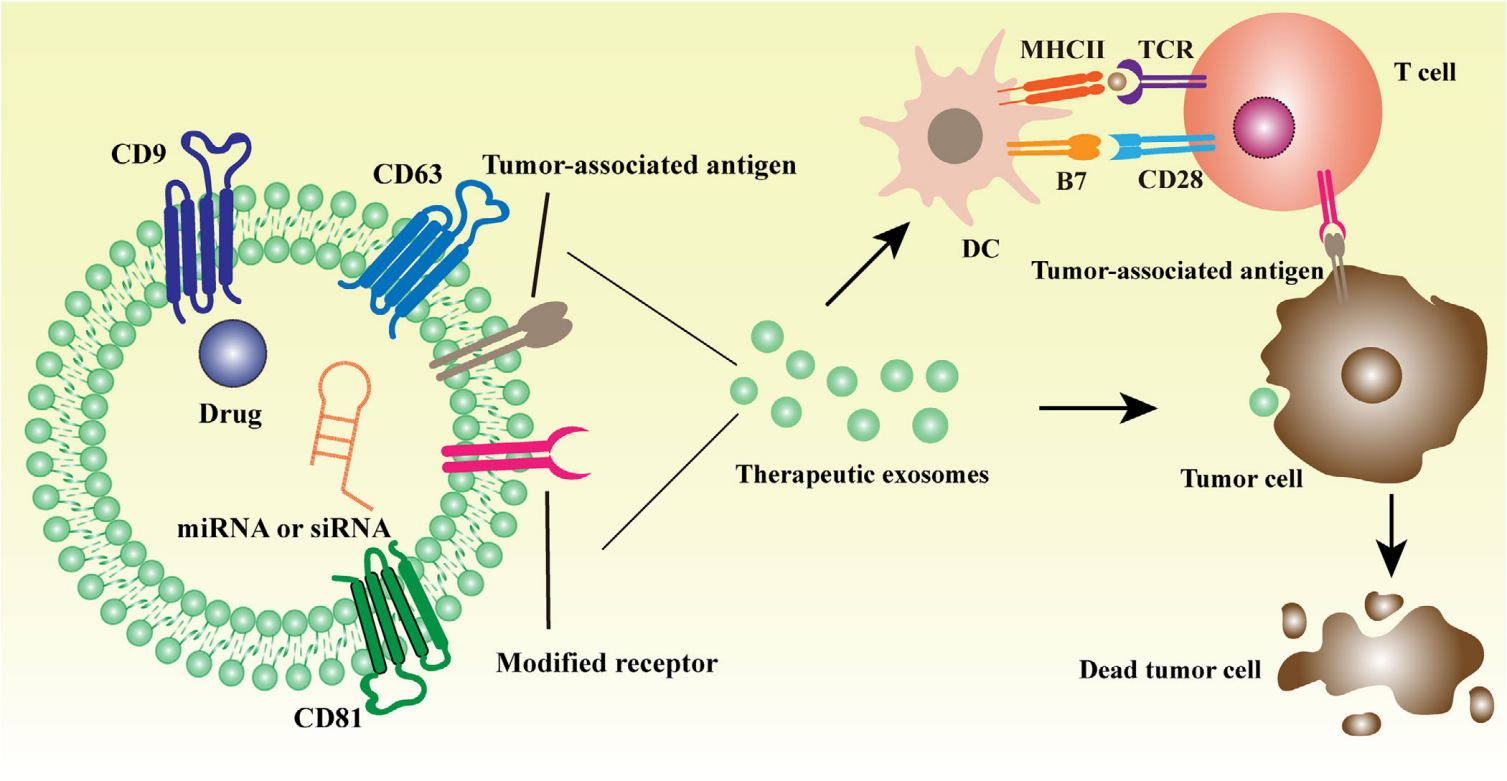
The mechanism of therapeutic exosomes on cancer. Therapeutic exosomes can be loaded with drugs, miRNAs, or siRNAs in vitro. The exosomes with drugs can be assimilated by tumor cell, then directly killing it. The exosomes carrying miRNAs or siRNAs also induce the death of tumor cell through changing the transcriptome, which affects the growth, proliferation even drug resistance of tumor cell. Furthermore, the surface markers of these exosomes can be modified in vitro to get better tumor targetability. The other kind of exosomes are used to enhance the immune response. Thereinto, exosomes can be used as the stimulator of DC vaccines to promote immune response. Exosomes expressing tumor‐associated antigen are able to stimulate DC. Thus, DC exerts its antigen‐presenting ability to activate T cell, subsequently kill tumor cell

With a deeper understanding of the remarkable loading capacity of exosomes, an exosomes‐based therapeutic strategy is not merely about the inhibition of their formation and secretion. Currently, exosomes are regarded as optimal drug delivery molecules because they have been proven to be well tolerated with unnoticeable side effects. Besides, exosomes are able to cross some barriers like the blood/brain barrier (BBB) or cytoplasmic membrane.^87^ Based on the observation that gene expression in recipient cells is repressed by exosomal miRNAs engaging target mRNA effectively, engineered exosomes carrying a specific small interfering RNAs (siRNA) or miRNA have been designed for cancer therapy.^88^ In addition, ligand/receptor modification on exosomes surface may also be applied to elicit or inhibit signaling events or targeting specific cancer cells^89^ (Table 2) (Figure 2).

**TABLE 2:** The role of exosomes for therapeutic use

The role of exosomes	Cargos	Cancer type	Mechanism of action	Ref.
Carrying chemotherapeutics	PTX	Prostate cancer, LC, BC	Increasing the cytotoxicity and decreasing the side effects of chemotherapeutics	92, 94, 137
	Doxil	CC, fibrosarcoma	Increasing the cytotoxicity of chemotherapeutics	91
	DOX	Glioma	Enhancing the permeability of crossing blood/brain barrier of chemotherapeutics	95
Carrying siRNA or miRNA	Sphk2 siRNA	HCC	Leading to the ablation of exosomal miRNA and the inhibition of the tumorigenic potency of TDEs	102
	miR‐335‐5p	HCC	NA	103
	miR‐374a‐5p	GC	Antagonizing Neurod1	104
	anti‐miR‐214	GC	Rescuing PTEN	105
	siKRAS	LC	Antagonizing KRAS	109
	circ‐Foxo3	BC	Promoting MDM2‐induced p53 degradation and increasing the expression of Foxo3 protein	106
Carrying both siRNA and chemotherapeutics	5‐FU and MiR‐21 inhibitor	CRC	Enhancing the cytotoxicity of 5‐FU and reversing drug resistance	112
	GRP78 siRNAs and sorafenib	HCC	Suppressing cancer cell growth, invasion as well as metastasis, and reverse the drug resistance	113
	MDR1 siRNA and PTX	OC	Enhancing the cytotoxicity of PTX	114
Stimulating immune response	SIRPA variants	CRC	Reinforcing the phagocytosis of phagocytes and promoting T cells' response	115
	Tumor‐associated antigens	GBM, HCC	As DC vaccines to induce the activation and proliferation of tumor‐specific cytotoxic T lymphocytes.	119, 122, 123

**Abbreviations**: BC, breast cancer; CC, cervical cancer; CRC, colorectal cancer; DOX, doxorubicin; Foxo3, forkhead box O3; GBM, glioblastoma; GC, gastric cancer; HCC, hepatocellular carcinoma; LC, lung cancer; MDM2, mouse double minute 2; OC, ovarian cancer; PTX, paclitaxel; TDEs, tumor‐educated exosomes.

### 5.1 Exosomes for carrying anti‐tumor drugs

Drug loaded into exosomes is a common strategy to enhance the efficacy, overcome the drug resistance as well as alleviate the side effects of chemotherapy and molecular target therapy. PTX incorporated into exosomes is delivered to multidrug‐resistant neoplasm and increases cytotoxicity nearly 50 times compared with PTX without exosomes delivery.^90^ TDEs present the preferable fused ability to their parent cancer cells. Therefore, Qiao et al and Saari et al injected back the TDEs loaded with Doxil and PTX beforehand in vitro, respectively, which showed a higher therapeutic efficacy compared with using drugs alone.^91^, ^92^ Bovine milk‐derived exosomes are proved to be an excellent carrier owing to non‐obvious side effects as well as high therapeutic efficacy.^93^ For example, bovine milk‐derived exosomes carrying PTX have been used as an oral medication to treat lung cancer. They not only show the significant inhibition of tumor growth but also have lower systemic toxicities compared with intravenous injection of PTX.^94^ In order to enhance the permeability of crossing BBB, focused ultrasound is developed for opening BBB in reversible and non‐invasive manners, which significantly inhibits glioma growth when combining with DOX‐incorporated exosomes.^95^ Despite the advantages of natural exosomes as drug carriers, there remain some challenges, such as high cost and low yield. One of the solutions is to develop a standardized technique for exosomes isolation, which can achieve large‐scale production and significantly reduce the contaminations.

Another solution is to find other alternative materials as drug carriers. Porous silicon nanoparticles and carbon nanoparticles are fantastic materials to carry chemotherapeutics and present significant cytotoxicity.^96^, ^97^ However, synthetic materials have a trouble realizing the biocompatibility of natural components. Inspired by exosomes, biomimetic nanoparticles recently have drawn considerable attention due to their potential to be potent drug delivery platforms. They combine synthetic nanoparticles with natural biomaterials including cell membranes or exosomes.^96^ Biomimetic nanoparticles have a series of merits, including excellent stability, superb loading capacity, inconspicuous toxicities as well as large‐scale preparative capability, which are ideal substitutes for natural exosomes and synthetic materials. A sort of biomimetic nanoparticles based on synthetic phospholipid bilayers is hybridized with membrane proteins from MCF‐7 cancer cells and red blood cells, which have homologous targeting ability and anti‐phagocytosis property, respectively. Compared with plain liposome, these biomimetic nanoparticles show higher tumor accumulation, lower interception so they possess a better antitumor therapeutic effect.^98^ Nanosponges and nanokillers (NSKs) are defined as a gold nanocage surface established with the platelet and neutrophil hybrid cell membrane. They exert their function of capturing circulating TDEs and cancer cells by the high affinity of membrane adhesion receptors. Thus, the communication based on exosomes between cancer cells is blocked. Furthermore, NSKs carrying with chemotherapeutics suppress not only the development of primary tumors but also the metastasis of BC.^99^

Exosomes can also be used as vehicles to carry CRISPR/Cas9 plasmids targeting tumor cells, which overcome the limitation of immunogenicity and low tolerance compared with traditional delivery vehicles, such as viral vectors and polycationic liposomes. CRISPR/Cas9 plasmids against PARP‐1 incorporated into TDE have been reported to decrease the expression of PARP‐1 and enhance the sensitivity to cisplatin in OC simultaneously.^100^

Drugs loaded into exosomes or biomimetic nanoparticles have a better performance possibly because they get access into cells more easily or they can deliver drugs into an optimal subcellular location of cells. In order to determine the exact mechanism, further research should emphasize the pharmacokinetics of these drug delivery vehicles, especially in vivo models. However, the visualization and trace of exosomes often require extensive sample purification and labeling in the pharmacokinetics studies, which faces technical difficulty currently. On the other hand, the big hurdle concerning exosomes as delivery vehicles is loading efficiency. Saari et al reported the loading strategy of co‐incubation only yielded 9.2% loading efficiency.^92^ The conventional electroporation is also limited by its low cargo loading efficacy while the protocol based on transfection reagent has high toxicity. Thus, a proper cargo loading strategy needs to be developed in the future.

### 5.2 Exosomes for carrying siRNA or miRNA

Generally, utilizing small RNAs (siRNA or miRNA) to regulate target protein is limited to the existence of some barriers in the human body, including cell walls, serum albumins, and blood RNases.^101^ The largest obstacle in the clinical translation of RNA‐based medicine is to find a safe and effective delivery system, which can deliver those small RNAs into the cytoplasm successfully. Lipids are the currently used vehicles for small RNAs delivery while liver toxicity is a barrier for clinical application. Recently, there is a growing number of studies focusing on the superior loading capacity and transferring competence to antagonize the oncogene or oncogenic coding RNA. As a checkpoint of Sphingosine‐1‐phosphate, sphingosine kinase 2 (Sphk2) may play a role in regulating the cargo loading into the exosomes. Liang et al designed Sphk2 siRNA‐loaded nanoparticles to reduce the expression of Sphk2 protein, ultimately leading to the ablation of exosomal miRNA and the inhibition of the tumorigenic potency of TDEs in HCC. It is found that the Sphk2 siRNA‐loaded nanoparticles can inhibit the tumorigenesis and the migration of HCC cells.^102^ Furthermore, exosomes loaded with miR‐335‐5p can be effectively taken up by HCC cells contributing to the inhibition of proliferation and invasion of HCC cells. The authors preliminarily identified 13 targets of miR‐335‐5p, among which casein kinase 1 gamma 2 and zinc Finger MYND‐type containing 8 were firstly reported to play a role in HCC. Unfortunately, the exhaustive mechanisms of miR‐335‐5p action have not been investigated in this study.^103^ MiR‐374a‐5p and miR‐214 inhibitors were incorporated into exosomes to reverse drug resistance by rescuing Neurod1 and PTEN in GC.^104^, ^105^ In addition to delivering miRNAs or siRNAs, exosomal circRNAs like circ‐Foxo3 begin to be applied to cancer therapy in recent years.^106^

For better targetability, the ligand/receptor on the exosome membrane is usually modified. A kind of engineered exosomes named synthetic multivalent antibodies retargeted exosomes (SMART‐Exos) express monoclonal antibodies CD3 and EGFR, which specifically targets T cells and cancer cells, respectively. SMART‐Exos cannot only connect T cells with cancer cells but also induce valid antitumor immunity both in vitro and in vivo.^107^ Folate receptor is a common cell surface maker in manifold cancers. Folate‐modified exosomes can promote targeted delivery of siRNA and enhance the loading competence by avoiding endosome trapping.^108^ A study revealed that folate‐functionalized exosomes from bovine milk carrying siKRAS can reduce 54% expression of KRAS compared with the control group.^109^ The high affinity and anti‐tumor efficacy highlight the novel strategy of using folate as a cancer‐targeted ligand for precisely treating cancer. Moreover, IL‐3R is overexpressed on chronic myelogenous leukemia (CML) blasts, so Bellavia et al coated a fragment of IL‐3 on exosomes to precisely target CML cells. It has been shown that engineered exosomes carrying Imatinib or BCR‐ABL siRNA are able to inhibit CML cell growth.^110^ Intriguingly, the membrane protein can be modified to enhance the loading efficacy of exosomes. MiR‐155 packed into exosomes, on which the C‐terminus of CD9 is combined with ELAVL1, is taken up by recipient cells in a more effective way.^111^ It is worth noting that the disturbance of surface protein on exosomes should be minimized when the exosomes are labeled. Furthermore, a simple construction platform should be developed to meet the demand for precision medicine, which allows the integration of various functionalities on exosomes.

In order to gain more significant anti‐tumor efficacy, the combination of gene editing and chemical treatment is usually packed into exosomes. MiR‐21i and 5‐FU simultaneously packed into engineered exosomes show the outstanding competence of reversing drug resistance, demonstrating high cytotoxicity of 5‐FU in CRC. Moreover, the combination of miR‐21i and 5‐FU within exosomes has a better performance in reducing drug resistance than using either single agent in recipient cells.^112^ Besides, GRP78 siRNAs coupled with sorafenib and multi‐drug resistance gene 1 (MDR1) siRNA with PTX were incorporated into exosomes to reverse drug resistance in HCC and OC, respectively.^113^, ^114^ Given that both siRNAs and miRNAs can be loaded into exosomes for gene editing therapy, it is necessary to compare their silencing effects. The latter are the endogenous molecules, which may reduce the off‐target effects compared with exogenous synthetic siRNAs. Additionally, miRNAs target a set of coding genes instead of a single one, suggesting it is likely to play a better role in overcoming drug resistance that refers to multiple molecular pathways.

### 5.3 Exosomes for stimulating the immune response

Exosomes have been designed as therapeutics for activating the immune response. TAMs play an important role in cancer development and drug resistance. Modulating TAMs through engineered exosomes is efficacious in tumor immune therapy. CD47, known as a “don't eat me” signal, presents on the membrane of most tumors and interacts with signal regulatory protein alpha (SIRPA) on phagocytes, then inducing the blockage of phagocytosis. Thus, Koh et al developed a sort of exosomes harboring SIRPA variants to reinforce the phagocytosis and promote T cells' response concomitantly. Strikingly, even low doses can exert the anti‐tumor function both in vitro and in vivo.^115^ Moreover, engineered exosomes from M1 macrophages modified with CD47 and SIRPA antibodies can abolish the “don't eat me” signal between macrophages and tumor cells. Meanwhile, these M1 macrophages‐derived exosomes can polarize macrophages from the M2 phenotype to the M1 one, achieving the additive anti‐tumor effect.^116^ Likewise, Choo et al reported that M2 macrophages can be repolarized to M1 macrophages by using exosome‐like nanovesicles from M1 macrophages, which was also able to enhance the efficacy of the anti‐PD‐L1 antibody.^117^ BC cells can retain dormant for decades for the most part in bone marrow stroma, which forms a vital niche for them to live in. These dormant BC cells are resistant to carboplatin, which are difficult to be detected and refractive to treatment in the early time, and this dormancy is partially caused by exosomes from M2 phenotype macrophages.^118^ Here, Walker et al found exosomes from M1 macrophages combined with carboplatin would make dormant BC cells resurgent and being sensitive to carboplatin via NF‐кB.^118^

In recent years, exosomes carrying tumor‐associated antigens have been the optimal stimulator for dendritic cells (DCs) vaccines. Although the exosomes as DC vaccines have achieved certain success, some problems pop out at the same time. It remains a difficulty in inducing a potent and durable antitumor immune response because of the insufficient tumor‐associated antigens carried by exosomes. Therefore, co‐delivery of TDEs and α‐galactosylceramide that is for strengthening antigens presentations induces the activation and proliferation of tumor‐specific cytotoxic T lymphocytes. This combination has shown the powerful effects in GBM immunotherapy because of the improvement of the immunosuppressive environment and the break of immune tolerance.^119^ The stimulator of interferon genes (STING) anchored in the endoplasmic reticulum acts as a cytosolic DNA sensor, which cannot be activated by double‐strand DNA directly. STING pathway is chiefly activated by the second messenger cyclic dinucleotide that is generated by cyclic GMP‐AMP synthase (cGAS). Stimulated by cytosolic DNA, the active cGAS‐STING pathway can promote the expression of type I interferon to facilitate DC maturation and T cells activation for initiating antitumor immunity.^120^ It is found that tumor cell‐free microparticles as an ideal vaccination platform can effectively transfer the tumor DNA fragment to DCs and lead to robust antitumor immunity, which is mediated by the cGAS‐STING signaling pathway.^121^ Besides, exosomes presenting can stimulate DC‐mediated immune response by regulating the secretion of cytokines.^122^ For instance, there are the reduced expression of IL‐10 and TGF‐β and more secretion of INF‐γ and IL‐2 accompanying with an increased number of T lymphocytes in HCC after the trigger of exosomal tumor‐associated antigens.^123^ But in PDAC, TDEs displaying abundant tumor antigens can induce humoral immunity. The autoantibodies tend to bind to exosomes, which play a bait‐like role, attenuating complement‐mediated cytotoxicity on cancer cells.^124^

## 6 CONCLUSIONS AND PERSPECTIVE

Drug resistance is still a big challenge in cancer therapy, and its underlying mechanism is not fully understood. In recent years, exosomes have been found to play a pivotal role in intercellular communication, including transmitting drug resistance traits via transporting vital nucleic acid or protein. As another category of EVs, microvesicles induce drug resistance in a similar mechanism as exosomes. Drug‐resistant cancer cells can expel chemotherapeutics or tumor suppressor miRNAs out of cytomembrane via microvesicles.^125^, ^126^ Besides, miRNAs or proteins transferred by microvesicles are also able to confer the resistant phenotype to sensitive cancer cells, in which P‐glycoprotein is the most popular research object about microvesicles‐mediated drug resistance.^127^, ^128^ Noteworthily, microvesicles begin to be applied to carry chemotherapeutics to reverse drug resistance of cancer.^129^ Large oncosomes (1‐10 μm diameter) are a relatively new classification of EVs, which were firstly reported in 2008.^130^ As for its role in drug resistance, it has been found that HSP‐enrich oncosomes play an important role in resistant‐associated secretory phenotype by the establishment of immune evasion.^131^ Atay et al provided an insight into the proteome of oncosomes derived from gastrointestinal stromal tumor to further offer a research orientation about its TKI resistance.^132^ However, the number of studies about the oncosomes‐associated drug resistance is still insufficient, and the relative mechanism remains to be exhaustively elucidated.

Although exosomes as nano‐delivery vehicles have shown outstanding capacity in cancer treatment, this field is still in its infancy and remains numerous challenges. The contents of exosomes are quite complex, and their functions are just starting to be elucidated. Furthermore, it is noting that apart from the signal responses induced by ex vivo exosomal RNAs, any exogenous RNA may be recognized as “foreigner” by recipient cells, resulting in the degradation mediated by RIG‐I, an RNA pattern recognition receptor.^1^ Besides, there remains concerned that exosomes gained from cancer cells may carry some oncogenic factors, which may facilitate tumorigenesis.^133^ Although mesenchymal stromal cells‐derived exosomes can significantly reduce the risk of tumor promotion compared with TDEs, more clinical trials should be conducted for evaluating the safety.^134^ In addition, future efforts should be focused on the precise identification and isolation of exosomes and the development of technologies to realize large‐scale preparation of therapeutic exosomes. Haraszti et al identified dilysocardiolipin as an essential component of exosomes and constructed artificial exosomes from purified lipid and protein components.^135^ Artificial exosomes with defined components may overcome the disadvantage of naturally isolated exosomes and have the advantage of standard production and facilitating the transition to clinical application.

## CONFLICT OF INTEREST

The authors declare that there is no conflict of interest that could be perceived as prejudicing the impartiality of the research reported.

## AUTHOR CONTRIBUTIONS

All authors contributed to drafting and revising the article, gave final approval of the version to be published, and agree to be accountable for all aspects of the work.

## AVAILABILITY OF DATA AND MATERIAL

Data sharing is not applicable to this article as no datasets were generated or analyzed during the current study.
